# Hospital and Institutional News

**Published:** 1912-05-18

**Authors:** 


### HOSPITAL AND INSTITUTIONAL NEWS.

### THE NEW CORONER FOR SOUTH LONDON.

Mr. Samuel Ingleby Oddie has been appointed Coroner for the South-Western District of London, at a salary of £1,150 a year, by the London County Council. The new coroner is, of course, the late Mr. Troutbeck's successor, the post carrying with it the coronerships of the City and Liberty of Westminster and the Duchy of Lancaster District (Savoy portion). When the vacancy was first advertised, The Hospital expressed very strongly the desirability that the office should be filled by a medical man, and we are pleased to see that the Council has adopted this suggestion. Mr. Oddie has had considerable experience both as a barrister and as a medical practitioner, while, as deputy coroner for the Western District of London since 1903, he has acquired considerable experience and knowledge of the duties of a coroner.

### HOSPITAL CRITICISM.

Our contemporary *The Builder* fails to recognise that its contention is that a layman's opinion can hardly be accepted as the last word on hospital construction *quâ* Sir Henry Burdett's criticism on the Sheffield Royal Hospital. We have not seen the Report of the Special Investigation Committee of this hospital which purports to rebut certain portions of Sir Henry Burdett's criticism, but the extracts from it in the Sheffield papers which we have seen do not completely, if at all, dispose of the practical criticisms on points of construction which Sir Henry's Report contains. If *The Builder* is to make its point effectual in this instance it must first publish a complete set of the plans of the Royal Hospital, Sheffield. Until this is done, having regard to the record and admitted up-to-dateness of the technical knowledge of the critic, *The Builder's* readers, like our own who understand the subject, will probably reserve their opinion until the whole of the plans are produced, or until they have had an opportunity of personally inspecting the buildings in question. The plans of the Sheffield Royal Hospital are the property of the trustees of that institution. We venture to suggest, in the interests of hospital construction and the institution itself, that the trustees should publish a complete set of the plans of the whole of this hospital, with sectional drawings, together with the Report of the Investigation Committee. If this suggestion be adopted, we hope a copy of the report, with the plans, will be sent to the professional and general press in due course.

### THE HINCKLEY ISOLATION HOSPITAL VINDICATED.

The special meeting of the Hinckley Hospital Committee, which we stated last week as about to take place to investigate the serious charges made against the staff, was held on Monday. The committee sat for two hours and a half, and it was decided on the motion of Mr. C. H. Parsons that the charges had not been proved. The satisfaction with which this result will be welcomed will be shared by our readers, who this week have the opportunity of hearing an authoritative view of the matter in the letter which we publish in another column from the matron, Miss Myra Forsyth. It is only necessary to add that the applications for some remuneration from Dr. J. A. T. Hall and the matron for their extraordinary services during the recent scarlet-fever epidemic have been granted.

### THE NEW HOSPITAL AT PUTNEY.

The new Putney Hospital, thanks to the munificence of Mr. Chester's will, will be opened free from debt at the end of the month. The ground floor is given up to the out-patients and casualty unit, with its waiting, consulting, *x*-ray rooms, and dispensary; and here, too, are the resident medical officer's and matron's quarters and the board room. In the central hall the genesis of the hospital is thus recorded: "Be it hereby gratefully placed on record that Henry Chester, of Putney, by his will bequeathed a sum of about £75,000, the income of which forms the original endowment of this hospital, and the site was the generous gift of Sir William Lancaster, J.P., of Putney." The site, indeed, is inviting, for it combines a view over Putney Lower Common with close proximity to traffic facilities. The theatre unit is elaborately designed, and the theatre itself has an entrance for patients through the anæsthetic room from the corridor. The lighting and ventilation deserve to be considered in detail, which we hope to do at an early date. There are four eight-bedded wards, two for men and two for women, a small ward for children, together with one containing two beds, which it is hoped may be reserved for paying patients. These wards are all on the first floor. Great pains have been taken with their decoration and equipment. A tiled dado, harmonising with the duck-egg Ripolin paint of the walls, is the colour scheme adopted. Lawson-Tait beds, terrano floors, electric lighting are details which give some idea of the equipment. Gas cooking is employed throughout. Large balconies have also been provided. The honorary secretary is Mr. Frank Taylor.

### THE DIVORCE COURT AS A BAR TO PUBLIC APPOINTMENTS.

A curious case has arisen with reference to the recent appointment of Dr. T. S. Goodwin, M.B., as medical officer to the No. 8 Hounslow District. Some weeks ago Dr. Goodwin was appointed to this post, subject to the taking up of references as to character. It was then discovered that his wife was suing him for a judicial separation. The Brentford Guardians considered this to be a disqualification and fresh advertisements were issued. These were responded to among others by Dr. Goodwin, who accompanied his application by a letter which regretted that a family misunderstanding had prevented his original appointment from being confirmed. Voting, however, took place, and Mr.

W. C. Tuke Johnson was elected. Hardly was the result declared when the clerk, Mr. W. Stephens, stated that he had received two letters, one from Dr. Goodwin and another from his solicitor, in which it was pointed out that the judicial proceedings had been withdrawn. Mr. W. A. Davies remarked that these letters ought to have been read before the vote was taken, and the clerk stated in reply that he could not read them without the permission of the Board. This excuse sounds exceedingly lame, since it appears doubtful if the majority of the Board knew till after the voting even so much as that a second letter had been received. This hole-and-corner way of treating a matter which may yet prove of great importance to Dr. Goodwin is exceedingly unsatisfactory, and Mr. Davies must be thanked for his persistence in bringing the facts, even thus late, to light. Apart from this, the index question of whether divorce court proceedings should be a bar to public appointments demands consideration. What are our readers' opinions?

### SELECT COMMITTEE ON SECRET REMEDIES.

Some interesting evidence was given by the witnesses before the Select Committee on Secret Remedies at last week's meeting. The first witness, Sir Nathaniel Highmore, Solicitor to the Board of Customs and Excise, explained in great detail the Medicine Stamp Duty Acts, mentioning that last year the revenue from the duty on proprietary and secret medicines was £327,826. In the last return made to the House of Commons the number of medicine stamps issued in one year was 41,757,575. This represents the sale in one year of over 41 million boxes or bottles of medicine, the majority of which were of the face value of 1s. to 2s. 6d. each; the statement gives an excellent idea of the vastness of the traffic in these articles in Great Britain. The second witness, Mr. Ledley, of the Privy Council Office, was examined at some length with regard to the Blue Book on Unqualified Medical Practice, which was compiled and published some time ago at the request of the General Medical Council. He said the Blue Book was merely a collection of opinions. As already pointed out, owners of proprietary medicines are taking great pains in the preparation of their case, and it is of the utmost importance that the case against secret remedies should be properly presented.

### HONOUR FOR A SCOTCH SUPERINTENDENT.

The University of Glasgow is to confer the degree of Doctor of Laws on Dr. Donald J. Mackintosh, M.V.O., M.B., Medical Superintendent of the Western Infirmary, Glasgow, who, it reminds the public, is "the author of various medical works, widely known as an expert in hospital construction and administration." Dr. Mackintosh, by the way, is understood to be actively advising the committee of the Chichester Infirmary with regard to the much-discussed, somewhat curtailed, and slow-maturing reconstruction scheme of that hospital. The term of discussion is now apparently passed, and within nine months, according to the contracts the stipulated time for completing, the work will have been concluded. If the realised scheme gives as much satisfaction as the hospital's recent financial improvement, Chichester Infirmary will doubtless be granted the prefix "Royal" for which it has often hoped.

### A CHEST HOSPITAL AND THE TUBERCULOSIS COMMISSION.

It was a happy touch of Dr. Siegmund Moritz, senior physician to the Manchester Hospital for Diseases of the Chest, to enliven the annual meeting by criticising the interim report of the Government's Committee on Tuberculosis in the light of the practice of his institution. The work of the proposed tuberculosis dispensaries is being done, as he said, at the out-patient department. The recommendations laid down in the report coincide, Dr. Moritz went on, with what has been done for years by the Manchester Hospital for Consumption. After outlining the after-care work by means of the Samaritan Fund, and through the generous help of Mr. Dunkerley, who has enabled certain patients to find more healthy employment on farms, Dr. Moritz declared that the only matter in which the new scheme differed from the old is that the Government's Committee recommends that the dispensary should be administered by a whole-time tuberculosis officer. There is no doubt that such a speech as this not merely prevents the annual meeting from falling into the rut of routine dulness, but does much to enlighten the public and the subscribers as to the value of the work which the chest hospitals have performed. The opportunity afforded by the annual meeting was taken advantage of with real skill in this case, and Dr. Moritz has done his institution a service which every other chest-hospital medical officer would do well to emulate. It should do much to secure the support of Manchester for the modernisation of the out-patient department and for the Röntgen-ray apparatus which Dr. Cunliffe has asked for.

### FATAL MOTOR-CAR ACCIDENT TO A DOCTOR.

The town of Wantage, Berks, has sustained a heavy loss by the tragic accident to Dr. Woodhouse, nephew of the late Sir Francis Laking. Dr. Woodhouse had been resident in Wantage for seventeen years and was much beloved among his patients and a wide circle of personal friends. He was driving his own motor-car with a lady accompanying him on the evening of April 27, when owing to a burst tyre the motor-car overturned and both were thrown out. The lady escaped with bad glass cuts on her face, but Dr. Woodhouse had his skull fractured, and after lingering some days he died. Among the many good deeds through which Wantage will long remember him was the enlargement of the Cottage Hospital carried out under his direction. This little institution, after passing through troubled times, is now a good specimen of its class, and is fortunate in being under the management of an excellent matron.

### MIDLAND HOSPITALS AND THE INSURANCE ACT.

We publish elsewhere the full text of some very important resolutions which were passed unanimously at a meeting of the Midland Hospitals which was held at the General Hospital, Birmingham, on Wednesday last week. Briefly it was agreed that as soon as the Insurance Act comes into operation, and provided that arrangements be made with the medical profession, insured persons applying for out-patient treatment should receive first-aid only when severe accident cases, treatment only when the mode required can only be carried out in a hospital, and a second opinion only on the recommendation of a medical officer on the panel. As regards insured persons admitted as in-patients the resolution contained the recommendation that a claim on behalf of the hospital will be made on the approved society of the local Insurance Committee, except possibly in cases where an in-patient ticket, as the hospital's regulations may require, has been produced. This would appear to be the technical formulation of that *pro rata* payment for which The Hospital has contended from the first, and the Midland Hospitals are to be congratulated upon having set a timely example in this matter.

### DR. JELLETT'S ATTACK ON THE INSURANCE ACT.

The most vigorous attack upon the Insurance Act from any class of hospital in the United Kingdom has perhaps been that of Dr. Henry Jellett, Master of the Rotunda Hospital, Dublin. We have previously noted the arguments which he has urged to show the disastrous effect which the Act if unamended will have upon the Rotunda's patients and also upon its famous training school. Dr. Jellett, however, has followed up his previous efforts by the publication of a special report to the governors. In this his former points as to the manner in which the Rotunda Hospital will be affected by the Insurance Act are driven home. Under the Act's provisions, he remarks, no payment can be made on account of sickness or maternity benefit in respect of any woman who is an in-patient during her confinement; and further payment of this benefit can only be made if the woman is attended during her confinement by a registered medical practitioner or certified midwife. By the first of these provisions all the in-patients and by the second the out-patients would lose their benefit. The effect, Dr. Jellett foresees, is that women so penalised will cease to use the charity. "The hospital, and with it the training school, will thus cease from lack of patients." These considerations apply to all maternity hospitals, and it is characteristic of a clear-sighted nation that they have been advanced first of all by a medical superintendent who is an Irishman.

### HOSPITAL DISPENSERS AND THE INSURANCE ACT.

With reference to the election of the National Pharmaceutical Committee on Insurance reported elsewhere, we again regret to notice that the authorities responsible for the formation of the pharmaceutical committees have not considered the demands of the Public Pharmacists' and Dispensers' Association for the election of at least one practising institutional pharmacist upon the committees. What is still more to the point is the fact that the calling of pharmacy will be perhaps the only representative body in connection with the Insurance Act that has not a lady representative upon its various committees. Surely there are plenty of competent lady pharmacists on the register. It is interesting to note, however, that Mr. Edmund White, one of the members selected, has had a large experience of hospital pharmacy, having at one time held the position of pharmaceutist to St. Thomas's Hospital. Although Mr. White was not selected as a representative of institution dispensers, his specialised knowledge and experience of that branch of pharmacy will be of great service to the Committee.

### A DISPENSER AND HIS HOBBY.

For many years we have published the relaxations and hobbies of hospital workers in a special column of their own. This week we note that of a hospital dispenser which is somewhat remarkable. Mr. F. Noad Clark, Pharmacist to the Paddington Board of Guardians at the Paddington Infirmary, rides his in the current issue of *Marvels of the Universe*. Written in a style which appeals to the popular taste, he tells the life-story of the argulus or fish-louse, and illustrates the whole with some photographs and micro-photographs of his own production. Mr. Clark has contributed largely upon the subject of micro-photography to the South London Entomological Society and to the Public Pharmacists' and Dispensers' Association, of which latter body he is a past-chairman and member of the council. He is the author of "Practical Hints on the Manipulation of the Microscope."

### THE FOOD OF MODERN SCHOOLBOYS.

We do not think that the schoolmasters have come very well out of the discussion on diet in schools which took place at the conference on the subject which was held at the Guildhall last Monday. Thus Mr. F. B. Malim, headmaster of Haileybury, who presided at the morning session, when "Diet as a teacher in physical, intellectual, and moral efficiency" was considered, made the astonishing statement that variety of food was difficult to secure when they were catering for 500 boys. If he had read Dr. Robert Jones' recent remarks in The Hospital on the varied diet given daily at Claybury Mental Hospital, where some 2,500 patients are catered for, Mr. Malim would have had additional reason to admit that school kitchens must be miserably behind hospital kitchens in the art of securing variety. It is the small, not the large, households which present this particular difficulty, for Mr. Malim cannot be taken seriously if he regards 500 as too small. After this it is not surprising that his experience is that schoolboys generally prefer the food cooked by themselves. His fears also of encouraging self-indulgence were in striking contrast to the views of Dr. Clement

Dukes, honorary consulting physician to Rugby School, and of Dr. Robert Hutchison, who affirmed that for schoolboys too much, and not less, is enough.

### LESSONS TO SCHOOLS FROM THE HOSPITAL KITCHEN.

The conference was apparently unanimous in the opinion that diet has practically no influence on character, a result not unexpected since there were few or no vegetarians present, and this is the class of dietetists which lays the greatest stress upon the psychological value of food. The medical lamentations of the necessity for school hampers inevitably enforce the conclusion that the generality of schools, and especially of public schools, is in urgent need of food reform, both as to quantity and variety; how urgent, we think, can be judged from the lame remarks of Mr. Malim, and confirmed beyond a doubt from the frank confession of the Rev. M. Pearson, of Ardingley College, that the question of cost was all important. The moral is that schools generally still continue, though to a less degree, the bad old custom of economising on the food which they supply to their pupils, and their admission of this is the most fruitful result achieved by the conference. The National Food Reform Association has done good service in getting this established, and medical and public opinion should now insist on a raising of the standard of diet in schools all the way round. From the hospital kitchen they have evidently much to learn.

### INDEPENDENT PRAISE FOR COLNEY HATCH MENTAL HOSPITAL.

The case of William Ball, which has been made the subject of a White Paper, has an interest for all mental hospital workers from the fact that the report of Sir George Savage, M.D., on his treatment in gaol expressly adds that subsequently on his removal to Colney Hatch Mental Hospital "he was kindly and properly treated." It is also satisfactory to have Sir George's assurance that Ball did not object to the apparatus with which he was fed at the hospital. It will be remembered that Ball was sentenced on December 22 to two months' imprisonment for wilful damage, and was certified insane on February 12. The White Paper referred to is the answer of the Home Office to allegations of ill-treatment, which has taken the form of a report from a member of the profession, chosen, at Mr. McKenna's request, from the Royal College of Physicians, well known not to be connected with the prison service. With the allegations against Ball's treatment in prison we have no concern, but, as the old public prejudice against mental hospitals is not quite dead, it is satisfactory that the public will learn Sir George Savage's high opinion of the patient's subsequent "kind and proper treatment" at Colney Hatch.

### THE STATE OF BRADFORD ROYAL INFIRMARY.

What might have developed into one of the customary complaints, which usually are fruitful of so little except friction, shows signs of promoting constructive proposals. A man who having met with an accident was turned away from the Bradford Royal Infirmary and passed on to the Workhouse Infirmary, because the hospital wards were not merely full but overcrowded, has since died. The inquest produced an explanation from Mr. Basil Hughes, house surgeon, who said that they were obliged to send patients to the Workhouse Infirmary for want of room. Mr. J. J. Barron, the secretary-superintendent, readily confirmed this at a subsequent interview, and stated that in one ward which was intended for thirty patients there were frequently thirty-five, and that in the men's ward eight patients were accommodated on mattresses placed upon the floor. The moral drawn by the house surgeon and by the secretary is the same: more beds must be provided in Bradford. This conclusion, it is instructive to note, is precisely that which was arrived at in the articles on "Complaints and their Investigation" which a hospital chairman contributed a few weeks ago to The Hospital. But to drive this point home to the public mind Mr. Barron's further statement must be quoted, that the Bradford New Infirmary Scheme appears to be at a standstill. Of the £200,000 required only £80,000 has been subscribed. Well, if the public of Bradford does not want patients to be turned away from the doors of its Royal Infirmary it must provide the balance of £120,000. It should provide something else as well: the motor ambulance for the rapid removal of patients without jolting from the scene of accident, as Mr. J. Aspinall Marsden, M.R.C.S., has had the good sense and happy promptitude to demand incontinently. The immediate gift of this ambulance by an individual would do much to restart public interest in the New Infirmary Scheme. Why the *Yorkshire Observer* should try to raise animus against the Royal Infirmary we are at a loss to discover. We invite our contemporary to study the article on "Complaints and their Investigation" mentioned above.

### THE LISTER MEMORIAL.

It is understood that the Royal College of Surgeons and the Royal Society are taking steps to form a committee to provide a memorial to Lord Lister. Invitations to join the committee will be issued shortly, when an appeal will probably be made public, together with the scheme or schemes which it is proposed to undertake.

### A CORONATION COTTAGE HOSPITAL.

Hospitals or extensions to hospitals in commemoration of the late King are, of course, very numerous, but so far the Coronation has not been very frequently the inspiration of such institutional progress. It is, therefore, interesting to note that the foundation-stone of the new Epping Cottage Hospital, which was laid last week by Lord Warwick, inaugurates an institution in memory of the Coronation of King George. The donor of the site is Mr. J. G. Pelly, and it is satisfactory to learn that about half the annual cost of maintenance has been promised.

### THE ROYAL PORTSMOUTH HOSPITAL'S FUTURE.

Not long ago we discussed in these columns certain features of the management and financial status of this institution (The Hospital, March 9, 16, and 23, 1912). We expressed the view that in both of those respects things might be improved upon; and although the Chairman of the Committee, Mr. Lapthorn, defended himself and his colleagues ably, it would be idle to say that he converted us to his own way of thinking. He quoted in his letter the Portsmouth *Evening News*, whose editor he described as a friendly but impartial critic. From this same organ we now learn that the future of the Royal Hospital is a pressing question. For a year or two, says an editorial article, the capacity of the hospital has proved inadequate to meet the requirements of the town. The feeling has long existed, so we read, that the hospital is not keeping pace with the development of the town. Recently a Hayling tradesman met with injuries which ultimately proved fatal; but he could not be admitted to the Royal Hospital, as it was full, and was sent in a jolting cab to the infirmary. And this usage of the infirmary as an overflow has evidently become not by any means an unusual event.

### HOW OUR VIEW HAS BEEN CONFIRMED.

"The Portsmouth Royal Hospital," proceeds the local paper, "is one of the best equipped of provincial hospitals; now, however, it seems to have reached a crisis which will require extraordinary measures, if the confidence and support of the public are to be held." The arrival of this crisis is being accelerated by the action of the Portsmouth Board of Guardians, who are getting a little impatient of the use made of their infirmary for accident and other urgent cases refused admission to the hospital. The infirmary, as they justly point out, is for the destitute, not for emergency patients who are not destitute. Out of humanity the Guardians have accepted many serious cases sent on from the Royal Hospital. The Board subscribes out of its funds one hundred pounds a year to the hospital, and is discussing seriously the question of withdrawing this subsidy. Without the least desire to be unfriendly to the Royal Hospital, or to increase its difficulties, we feel constrained to point out that a falling revenue, accommodation already insufficient to meet local needs which are constantly increasing, and dissatisfaction expressed by the local press are signs confirmative of what we wrote in March. If the committee of management could be persuaded to adopt our suggestions, we feel sure that the local predictions of rate-aid would soon be falsified.

### A DISPENSARY AND CINEMATOGRAPH SHOWS.

The Brixton Dispensary, which was founded in 1850 by the late Dr. Wray, has recently been offered a chance of benefiting from the profits of the Sunday takings of a local cinematograph theatre. The amount of the assistance is estimated at about £200 annually. But the committee is threatened, if it accepts this proposal, with the resignation of five vice-presidents—local clerics—and a withdrawal of their Church aid, amounting to over £70 annually, which has hitherto been freely given. The Hospital Sunday Fund donation of over £40 will also be affected.

### WHY HOSPITALS MUST NOT BENEFIT FROM SUNDAY CINEMA SHOWS.

This is worth quoting because it shows that however large or small the gains offered may be, hospitals and dispensaries are always brought into trouble, or, what is worse, bring other and larger voluntary agencies into trouble, by seeking to benefit from Sunday cinematograph shows. The case in favour of them is obvious enough, but we have always argued with unimpeachable logic that so long, and we hope it will be for ever, as the voluntary support of hospitals in this country is dependent upon Hospital Sunday and a large general measure of Church support, so long must the hospitals refrain from offending Church support, or take upon themselves the very grave responsibility of inciting disaster to the Sunday Fund Movement. Where contributions from such shows have been accepted, a movement similar to this at Brixton for the withdrawal of Sunday Fund and Church support has always resulted, and this must be stopped by every hospital sufficiently imbued with the voluntary spirit to see beyond its own immediate gain. The rights and wrongs of the Church objection to Sunday cinematograph shows as such is a matter of opinion which does not affect this principle of solidarity at all.

### THE EVOLUTION OF A POOR-LAW INFIRMARY.

The opening of the new infirmary and other alterations to the Hinckley Workhouse have given what is virtually a new and important institution to Leicester. Of the four blocks formed by the additions and alterations the new infirmary is one, the new kitchen another, and the laundry a third. The infirmary is designed to accommodate forty patients, which are divided among two wards, with eighteen beds and a separation ward, and contains day rooms and lavatory accommodation for both sexes, while the nurses' rooms are on the floor above. The Rev. A. E. D. Disney, chairman of the Board of Guardians, recalled at the opening how small a part the infirmary played when the workhouse was first founded in 1837, although it was designed for 400 inmates even then. In those early days there were only sixteen beds. The Local Government Board has not been sparing of inducements for the increase of this accommodation, and in 1902 £300 was spent on improving the infirmary. The Board, however, refused to regard this as anything more than a temporary measure, and in 1908 Mr. W. T. Grewcock's plans were accepted. The Local Government Board, suspecting dilatoriness, then caused a surprise visit to be made, and after a home for children had been erected at Burbage this new infirmary has in turn been built. It has cost, including the new laundry and other alterations, £5,900. Mr. John Burns' Department is sometimes abused like all Government offices, but the evolution of this Poor-Law infirmary, so well outlined by Mr. Disney, shows that infirmary progress often owes much to the vigilance of the Local Government Board.

### EVERY DESIGN RETURNED!

ARCHITECTS who submitted competitive designs for a cottage hospital at St. Anne's-on-Sea have had their designs returned with a comment that a competent assessor has advised that not one of the designs can be erected for £2,500, the limit specified. It would be interesting to know what accommodation the promoters suggested. Till that is established little can be said, though, in the event of the committee having demanded more accommodation than the cost specified can allow, the assessor has done wisely in pointing this out now, and so protecting the architects from later criticism.

### HONORARY STAFFS FORTY YEARS AGO.

DR. ROBERT SAUNDBY, who has been honorary physician to the Birmingham General Hospital for twenty-seven years, was the recipient of a presentation last week. The address which accompanied it and was handed by Sir Robert Simon, senior physician, to the Lord Mayor, Alderman Bowater, for presentation to Dr. Saundby is signed by the ninety-five subscribers to the special fund. In his reply Dr. Saundby recalled that when he first entered the hospital's service thirty-six years ago Mr. Timothy Kenrick was chairman of the board, while to-day only his friends Mr. Joseph Burrows and Mr. John Phillips remained of those who formed that body. But the changes in the board, great as they were, were not so great as those shown by the hospital. He hoped that those who were beginning their work there would at the end of thirty or forty years be able to look back on as much progress as he, especially in the growth of that spirit by which every member of the staff now devoted to the work of the hospital, not merely the time he could spare from his private practice, but his time, strength, and energy, regarding the work at the hospital as his first call. Mrs. Saundby was presented with a necklace in recognition, to quote Sir John's Holder's words, of the splendid work she has done on the Samaritan Committee.

### A NEW OUT-PATIENT DEPARTMENT IN RETROSPECT.

MR. DOUGLAS OWEN, in moving the adoption of the annual report of the Paddington Green Children's Hospital last week said that the institution was not one penny in debt over the new out-patient department, which was added in October last. Indeed the board hopes presently to be in the same position as it was before anything was spent on the new, or rather reconstructed, building. Of course, so extensive an addition means an increased annual expenditure, but the members of the board are very confident with regard to the future. Mr. Owen also drew attention to the proposal to provide picture tile panels—the growing popularity of which, as a form of hospital decoration, we had occasion to point out in a recent issue—as special gifts to the new department by friends of the hospital, and he hopes that those who have an interest in the institution will see their way to provide them. At the same meeting an interesting speech was made by the Rev. E. P. Anderson, who deplored the decline of outside agencies in regard to hospital collections. The new out-patient department has evidently been a success, but why has there been a delay in appointing an almoner?

### AMBULANCE WORK IN INDIA.

THE widespread interest in first-aid and home nursing which is displayed in Great Britain is due mainly to the establishment in 1877 of the Ambulance Association of the ancient Order of St. John of Jerusalem in England; and it has been stimulated in the last four years by the devolution upon Voluntary Aid Detachments of the responsibility for the care and transport of the wounded, under the Territorial scheme, between the field ambulances and the general hospitals in large towns. Nor is it in Britain alone that this work is extending. In India there is a branch which is rapidly extending its organisation and utility. Lately a monthly paper has been started to assist in disseminating a knowledge of the movement and of first-aid principles in general. In this work native Indians are sharing, as well as European officers and surgeons. The Viceroy is President of the branch, and thirty centres are now listed. Text-books in various Indian languages have been published, and the evidence is clear of the vitality of the movement.

### THIS WEEK'S DRUG MARKET.

FEW important changes in the prices of drugs have taken place during the week, but, on the whole, business continues good. Santonin has again been advanced in price, the advance being about 9s. per pound, which is double the amount of the usual addition; there is nothing to indicate that the highest figure has as yet been reached by this article, but in view of the extremely high price now quoted, it would hardly be wise for purchasers to buy more than sufficient to meet their immediate requirements. There has been no further rise in the price of quinine, and during the past week the demand has been quiet. Opium is unchanged, with a downward tendency in price; morphine also tends lower without any quotable change. Makers of codeine have announced a reduction in their quotations for this alkaloid. Oil of cloves is again slightly dearer. Ether and collodion tend slightly higher in price owing to the advance in the cost of German spirit. Carbolic acid tends slightly downward in value. Balsam tolu is again rather dearer. It is anticipated that the English crops of henbane and belladonna will be small owing to absence of rain last month.

### TO OUR READERS.

CONTRIBUTIONS are specially invited from any of our readers to these columns. They should deal with topical subjects and news. They must be authenticated for the information of the Editor only. The minimum payment if published is 5s. There is no hard-and-fast rule as to space, but notes of about twenty lines in length are preferred.

